# Digital technologies for mental health improvements in the COVID-19 pandemic: a scoping review

**DOI:** 10.1186/s12889-023-15302-w

**Published:** 2023-03-01

**Authors:** Jinhui Li

**Affiliations:** 1 School of Journalism and Communication, Jinan University, 601 Huangpu Ave West, 510632 Guangzhou, Guangdong China; 2 National Media Experimental Teaching Demonstration Center, Jinan University, 601 Huangpu Ave West, 510632 Guangzhou, Guangdong China

**Keywords:** Internet-based intervention, Emotional well-being, Coronavirus disease, Psychological treatment

## Abstract

Digital technologies have been used to support mental health services for two decades, but the COVID-19 pandemic created a particular opportunity for greater utilization and more data-driven assessment of these digital technologies. This research aims to offer a scoping review of the characteristics and effectiveness of digital interventions that were employed to improve mental health in the real context of COVID-19 pandemic. A combination of search terms was applied for automatic search of publications in the relevant databases. The key features of included studies were extracted, including the intervention, participant, and study details. A total of 20 eligible studies were included in the final review, which were conducted across different geographic regions and among diverse cultural groups. Among them, fourteen studies mainly reported the impact of digital technologies on general population, while only one published study developed specific interventions for the isolated COVID-19 depressed patients in hospitals. Digital technologies identified in this review were mainly developed via web-based and mobile-based platforms, such as social networking and video conferencing applications. But less than half of them were aligned with theoretical approaches from standardized psychological treatments. Most of the studies have reported positive effects of digital technologies, either on improving general mental and emotional well-being or addressing specific conditions (e.g., depression, stress, and anxiety). This scoping review suggests that digital technologies hold promise in bridging the mental health-care gap during and after the COVID-19 pandemic, and calls for more rigorous studies to identify pertinent features that are likely to achieve more effective mental health outcomes.

## Introduction

The coronavirus disease 2019 (COVID-19) pandemic and the consequent measures, such as social distancing and global lockdown, have posed a significant challenge for both individuals and public. Empirical evidences from both developed and developing countries have identified increased burden of mental health-care system during the COVID-19 pandemic [1–3]. For instance, negative psychological impacts were observed in the population due to the quarantine, including increased incidence of depression, perceived stress, and loneliness [4]. Because of the public health measures, the reduction of in-person mental health services and limitations in patients’ mobility further deepened the existing shortcomings of mental health care systems [5]. In light of the current situation, several studies [6, 7] have called for an urgent need to develop and adopt the emerging digital technologies in order to address gaps in mental health services during and after the global pandemic.

Digital technologies offer effective and timely solutions that scale-up and decentralize health care across a wide variety of platforms, from teletherapy, mHealth (mobile health) applications, to web-based interventions. They have been used to support mental health services for more than two decades, but the COVID-19 pandemic creates a unique opportunity for greater utilization and more data-driven assessment of these digital technologies in combating the pandemic-driven surge in mental health disorders [7]. Because of the increased demand, an overview of how the digital health tools could assist in reducing the mental health problems of COVID-19 is important, to provide comprehensive recommendations for future application of digital mental health interventions during and beyond public health crisis. Some recent rapid reviews [8, 9] were conducted under the developing situation – however, they only involve a general description of potential digital interventions that may help to address the mental health issues of the COVID-19 pandemic. Evidence-based synthesis of the actual implementation techniques and effects of digital mental health technologies during public health crises, particularly this ongoing pandemic, is currently lacking. As World Health Organization highlights, “rigorous evaluation of digital health is necessary to generate evidence and promote the appropriate integration and use of technologies” [10]. This research thus aims to provide a comprehensive and systematic scoping review of the characteristics and effectiveness of digital interventions which were employed to improve mental health in the real context of COVID-19 pandemic.

## Methods

### Search strategy

The current scoping review followed the guidelines from Preferred Reporting Items for Systematic Reviews and Meta-Analyses (PRISMA) [11] with minor modifications made where appropriate, to ensure clarity and transparency of review reporting. With an aim to identify digital technologies which have been applied specifically for the mental health problems occurred during the period of COVID-19 pandemic, a search was performed on 14 February 2022 by consulting the following databases: IEEE Digital Library, ACM Digital Library, PsycINFO, PubMed, and Web of Science. A combination of search terms was applied for the automatic search of publications in the above databases, including (“digital” OR “technology” OR “e-health” OR “tele*” OR “internet” OR “online”) AND (“mental” OR “psychological” OR “emotional” OR “depress*” OR “stress”) AND (“COVID-19” OR “coronavirus” OR “pandemic”). Reference list of the included studies or relevant reviews was further inspected for additional articles.

### Selection criteria

**Fig. 1:**
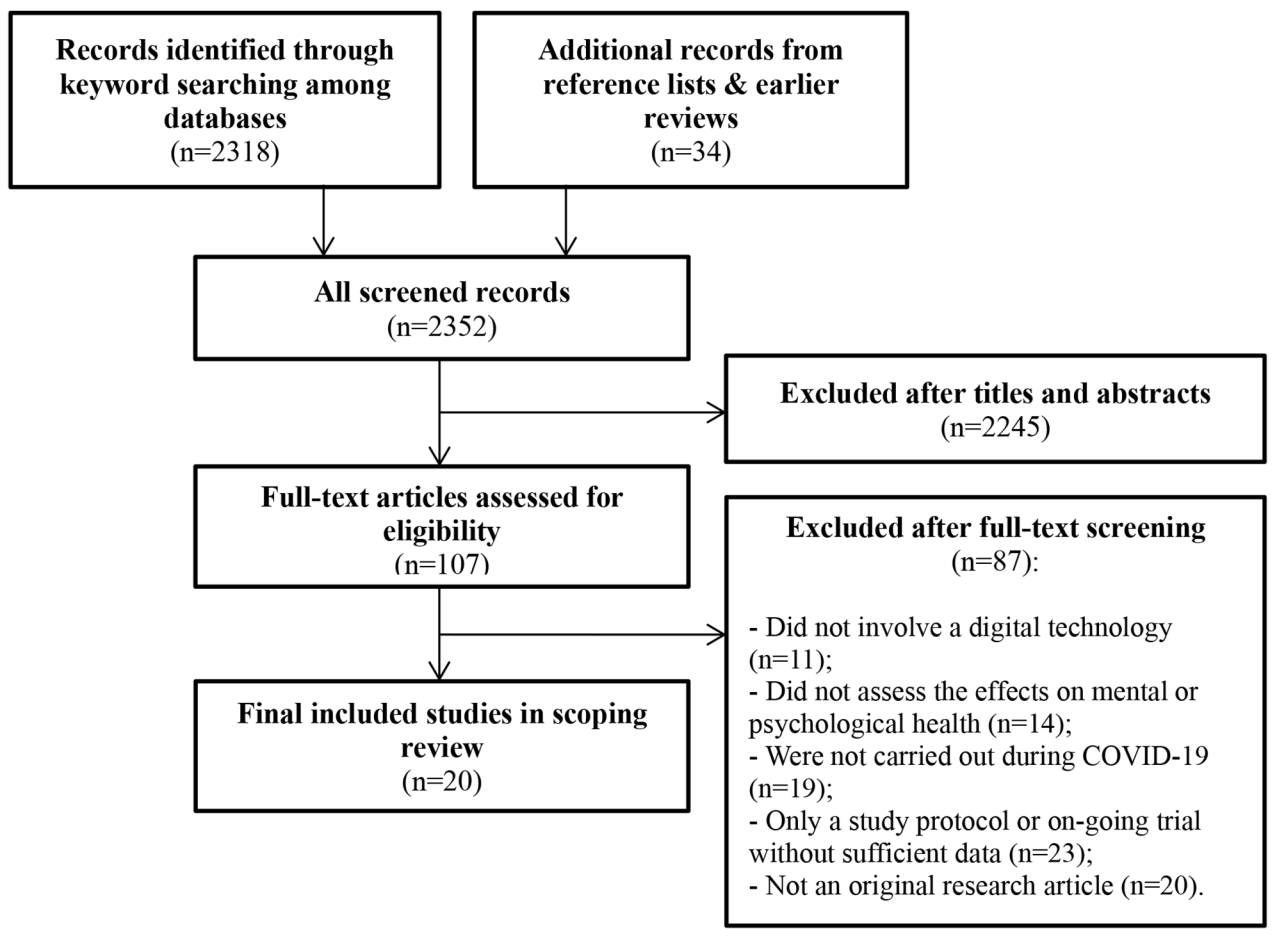
Flowchart for the included studies in the scoping review

In the screening process, titles and abstracts were first assessed for inclusion, following by the full text to determine the final included studies in the scoping review. For the inclusive criteria, eligible studies had to be carried out during the COVID-19 pandemic, which aimed to evaluate the effectiveness of a digital health intervention on mental health needs or problems took place in the COVID-19 context. For the purpose of this review, we adopted the classifications from WHO [12] and defined the target term “digital health interventions” as “the different ways in which digital and mobile technologies are being used to support health system needs”. They therefore include Internet, mobile applications, video game consoles, sensors and other relevant telecommunication tools. In addition, we only included journal articles published after 2019 when COVID-19 first occurred. However, due to the small number of potential studies, there is no limitation in terms of participant types, study design and mental health outcomes (i.e., depression, stress, loneliness, etc.). Those without primary evaluation data were excluded, such as study protocols, on-going trials, commentaries, or reviews. The initial search yielded 2,352 papers, of which 20 studies were included in the final scoping review. The selection process is demonstrated in the Fig. 1.

## Results

Key characteristics of the 20 included studies were first extracted, including the intervention features (platform and techniques, offered functions), participant information (country, sample size, age, and profile), as well as study details (methodology, outcome measures, and major findings). Table 1 outlines the extracted characteristics of these studies. As expected, all articles were published after the year of 2020, with eleven of them published in 2021 and two in 2022.

**Table 1:** Summary of the included studies

Study	Participant				Intervention		Study		
	Location	Age	Eligibility criteria	Sample size (completed)	Platform and techniques	Offered functions	Methodology	Mental health Measurement	Mental health outcomes
Agyapong et al. [28]	Canada	N.A.	N.A.	766	SMS text messaging	Providing daily supportive messages based on CBT	Web-based survey; Pre- and post-measurement; 6 weeks	Stress (PSS-10), Anxiety (GAD-7), Depression (PHQ-9)	Statistically significant reductions in: - Stress (t = 3.99, p < .001) - Anxiety (t = 9.86, p < .001)
Boucher et al. [14]	United States	18–64 years	Adults experiencing loneliness	11	Gamified mental health intervention	Providing gamified versions of evidence-based therapeutic activities	Asynchronous focus group; 3 days	Loneliness (Open-ended discussion and questions)	More active coping strategies (qualitatively) to address loneliness
Brog et al. [26]	Germany	> 18 years	Adults with at least mild depressive symptoms	107	Internet-based CBT intervention	Providing psychoeducational training	RCT study with waiting control group; Pre- and post-measurement; 3 weeks	Depression (PHQ-9), Psychological distress (DASS-12), emotion regulation (SEK-27), Loneliness (UCLA Loneliness Scale), Resilience (RISC)	Statistically significant increase in: - Emotion regulation skills (d = 0.35) - Resilience (d = 0.38)
Bureau et al. [22]	France	19–55 years	Healthcare workers	10	Internet-based CBT intervention	Providing psychoeducational training	Web-based survey and phone interview; 1 week	Perceived stress (phone interview)	Reductions (qualitatively) in perceived stress
Charbonnier et al. [21]	France	N.A.	University students	114	Online self-help program (via Facebook)	Providing psychoeducational training	Non-randomized controlled study with control group; Pre- and post-measurement; 8 weeks	Anxiety and depressive symptoms (HADS), Learned helplessness (LHQ)	Statistically significant reductions in: - Anxiety (rrb = 0.49) - Learned helplessness (rrb = 0.51)
Deng et al. [19]	China	18–22 years	University students	1607	Web-based physical education	Providing web-based sports education during quarantine	Web-based survey	Depression, Anxiety, and Stress (DASS-21)	Statistically significant reductions in (compared to a previous study): - Depression - Anxiety - Stress
Ellis et al. [13]	Global	> 18 years	Adults playing certain AR games	2004	Location-based augmented reality (AR) games	Promoting increased physical activity and social connection	A mixed methods web-based survey	Mental well-being (WHO-5, and qualitative questions)	- General improvement on mental health (77.20% participant reported); - Improved emotional coping (42.60% participants reported)
Firdhous [25]	Sri Lanka	> 18 years	Adults using online social media regularly	231	Online social media network	Maintaining the social contacts with friends and relatives	Web-based survey	Social resilience (Single item question)	Reduction in boredom (79.22% participants reported)
Gabrielli et al. [27]	Italy	18 to 34 years	University students	71	Chatbot supporting healthy coping	Providing psychoeducational training	Web-based survey; Pre- and post-measurement; 4 weeks with two per week	Perceived Stress (PSS-10), Anxiety (GAD-7), Mindfulness (FFMQ)	Statistically significant reductions in: - Anxiety (t = 0.94, p = .009); - Stress (t = 2.00, p = .05)
Goodman-Casanova et al. [18]	Spain	> 60 years	Community-dwelling older adults with mild cognitive impairment or mild dementia	93	Television-based assistive integrated service	Providing remote support through data transmission and video interactivity between users and caregivers	RCT study with control group of receiving treatment as usual	General mental health (Quantitative and open-ended questions)	No significant improvements in mental health
Li et al. [29]	China	> 18 years	None	1530	Internet hospital consultation (via WeChat)	Providing free internet medical consultations	Web-based survey; Pre- and post-measurement	Psychological stress (GHQ-28), Concerns about COVID-19 (Author-generated quantitative questions)	Statistically significant reductions in - Psychological stress (number of participants: χ^2^ = 1704.80, p < .001); - Degree of concern (t = 90.64, p < .001)
Loveys et al. [16]	New Zealand	> 18 years	Adults with an underlying medical condition or aged > 70 years with MMSE score > 24	24	Digital human facilitator (conversational agent with artificial intelligence) on website	Providing cognitive behavioral and positive psychology exercises	RCT study mixed design with waitlist control group; Pre- and post-measurement; 15 min per day over 1 week	Loneliness (UCLA Loneliness Scale); Psychological stress (PSS-4), Worry about contracting COVID-19 (Single item question); Psychological well-being (8-item Flourishing Scale)	No significant improvements in mental health
Kawakami et al. [24]	Japan	20–59 years	Adult employees	902	COVID-19 Contact Tracing App	Notifying when coming into close contact with a person with positive COVID-19 test	Web-based survey; Pre- and post-measurement	Worry about COVID-19 (single-item scale), Psychological distress (K6 scale)	Statistically significant reduction in psychological distress (associated with downloading app: OR = 0.61, 95%CI = [0.39–0.93], p = .02)
Pizzoli et al. [31]	Italy	> 18 years	Adults without any impairment of auditory abilities	240	Web-based relaxation practices	Offering web-based natural sounds, deep respiration, and body scans	RCT with three experimental conditions: a guided square breathing exercise, a guided body scan exercise, or natural sounds; Pre- and post-measurement; 7 min	Perceived relaxation (VAS), Emotional states (SAM)	Statistically significant improvements (of all three interventions): - Perceived relaxation (p < .001) - Psychomotor activation/stress (p < .001) - Fear related to COVID-19 (p < .001)
Ruiz-del-Solar et al. [23]	Chile	15–83 years	Isolated COVID-19 patients	Two hospitals; 986 visits	Telepresence robot	Assisting health-care workers in providing mental and psychological health services	Qualitative field study; 8 weeks	Mental and emotional health problems (Nonparticipatory and participatory observation, field notes, structured interviews and anecdotal records)	Increase of positive emotions (qualitatively) in patients and health-care workers
Sharrock et al. [30]	Australia	> 18 years	N.A.	1295	Internet-based CBT intervention	Providing psychoeducational training through the story of a fictional character	Web-based survey; Pre- and post-measurement	Health anxiety (SHAI), Depression (PHQ-9), Psychological distress (K-10)	Statistically significant reduction in: - Health anxiety (g = 0.89) - Psychological distress (g = 0.91) - Depression symptoms (g = 0.55)
Shapira et al. [17]	Israel	> 65 years	Community-dwelling older adults with internet access	82	Digital group intervention via Zoom	Providing online guided sessions in small groups	A pilot-RCT study with a wait-list control group; Pre- and post-measurement; 7 weeks with twice-weekly	Loneliness (UCLA Loneliness Scale), Depression (PHQ-9)	Statistically significant reduction in: - Loneliness (F(1,78) = 5.59, p = .02) - Depressive symptoms (F(1,78) = 0.57, p = .45)
Song et al. [20]	China	> 18 years	Adults with depression symptoms (PHQ-9 score: 5–27); Access to smartphone	129	Mobile application based on WeChat	Providing self-help storytelling to help users overcome mental health problems related to COVID-19	RCT with a wait-list control group; Pre- and post-measurement; 1 week with three sessions	Depression (PHQ-9), Anxiety (GAD-7), Insomnia (ISI), Psychological resilience (RISC), Anxiety of COVID-19 (VAS)	Statistically significant reduction in: - Depression (F = 4.30, p = .040) - Insomnia (F = 7.10, p = .009)
Stuart et al. [32]	Australia	Average age = 23.03 years	N.A.	473	Socially motivated Internet use (online social connection)	Providing online social connection	Self-reported survey	Depression (DASS)	Statistically significant reduction in depression (three-way interaction between health anxiety, isolation behaviors, and Internet use for social connection: β = -0.12, p = .009)
Summers et al. [15]	United Kingdom	22–70 years	N.A.	347	Digital behavior change app	Providing educational and therapeutic behavioral change support	Open-label survey; Pre- and post-measurement; 12 weeks	Anxiety (GAD-7), Depression (PHQ-9), Perceived stress (PSS)	Statistically significant reduction in: - Depression (t(272) = 15.60, p < .001) - Anxiety (t(272) = 15.90, p < .001) - Perceived Stress (t(272) = 22.40, p < .001)

Note: DASS-21, Depression, Anxiety, and Stress Scale; FFMQ, Five-Facet Mindfulness Questionnaire; GAD-7, Generalized Anxiety Disorder–7 scale; HADS, Hospital Anxiety and Depression Scale; IAS, Illness Attitude Scale; ISI, 7-item Insomnia Severity Index; K6, Kessler 6 scale; LHQ, Learned Helplessness Questionnaire; MMSE, Mini-Mental State Examination; PHQ-9, Patient Health Questionnaire–9; PSS-10, 10-Item Perceived Stress Scale; RCT, Randomized Controlled Trial; rrb, rank biserial correlation; SAM, Self-Assessment Manikin; SEK-27, Self-report Measure to measure emotion regulation skills; SHAI, Short Health Anxiety Inventory; RISC, Connor Davidson Resilience Scale; VAS, Visual Analogue Scales; WHO-5, World Health Organization–5 Well-Being Index

### Target population

In terms of location, the identified digital technologies were adopted and assessed across different geographic regions and among diverse cultural groups. Particularly, more studies were published from Asia-Pacific (eight studies) and European countries (eight studies), while less from other regions such as North America (two studies) or South America (one study). One study [13] recruited participants from over 60 countries, yet half of which were from the United States. Most of the studies targeted adult individuals aged above 18 years. Some of them reported the upper threshold such as 64 years [14] or over 70 years [15, 16]. Two studies particularly evaluated the effect of digital technologies on community-dwelling older adults [17, 18], while three studies focused on young generation such as college or university students [19–21]. Additionally, the sample size did vary a lot across the included studies. For example, four studies involved a large number exceeding 1000 participants (the largest one is 2004 participants from Ellis et al. [13]), while four studies included a small sample of less than 100 participants (the smallest one is 10 participants from Bureau et al. [22]). Ruiz-del-Solar et al.’s study [23] reported the number of visits (a total of 986 visits) which have been made to patients using digital intervention, rather than the actual participant number.

Besides the difference in age and sample size, participants from the included studies were further recruited with various inclusive criteria. For example, the samples of these studies varied a lot in occupation, such as college students [19], healthcare workers [22] or employees [24]). Some studies involved criterion regarding media interaction, such as using online social media regularly [25] or having played certain mobile games [13]. Besides general population, some digital technologies were adapted to people with certain mental problem, such as loneliness [14], depression [26], or cognitive impairment (e.g., mild dementia [18]). One study from Ruiz-del-Solar et al. [23] further reported a specific intervention for the isolated COVID-19 patients within the hospital context.

### Description of digital technologies

Based on the platform technology, three types of digital mental health interventions were identified in the current scoping review: web-based, mobile-based, and external device-based interventions. More than half (12 studies) of the digital technologies were mainly developed on web-based platforms, demonstrating their popularity in delivering mental health therapies and services during the COVID-19 pandemic. For instance, several self-help learning programs [22, 26] or chatbot supporting interventions [16, 27] were implemented and delivered through Internet, to enhance people’s coping skill for mental health problems during the COVID-19 pandemic. Specific social-networking or video-conferencing applications were also used to provide psychoeducational trainings, such as Facebook [21] or Zoom [17]. Another common type of digital technologies in this review is mobile-based interventions. Agyapong et al. [28] introduced “Text4Hope”, a daily supportive text message program in Canada, with the aim to provide convenient, cost-effective, and population-level interventions during the COVID-19 pandemic. Two studies [20, 29] further described the use of WeChat (a Chinese instant messaging mobile application) to provide medical consultation and education regarding mental health problems related to COVID-19. Besides using the existing platforms, there were two interventions built on external digital devices: one is a television-based assistive integrated technology developed for remote support [18], while another is a social robot “Pudu” to be used in hospitals [23].

Among all the digital interventions, it is important to notice that less than half of them [14, 17, 20–22, 26–28, 30] reporting adapted contents and activities from cognitive behavioral therapy (CBT), positive psychology, or other relevant frameworks. In other words, they were aligned with theoretical approaches from standardized psychological treatments. For the remaining interventions, they did not report an explicit psychological framework to support the intervention development, despite that some of them also provided effective tools (e.g., online consultation [29], web-based training [31], or remote support [18, 23]) to reduce psychological burden associated with COVID-19. Several digital technologies were not primarily designed for promoting mental health care, such as location-based augmented reality game [13], web-based physical education [19], and government-issued COVID-19 contact tracing app [24] – but researchers simply evaluated their effects on mental health problems during the pandemic. Furthermore, some digital technologies involved additional functions or services that were closely related to present circumstances of the pandemic, including promoting health education and knowledge of COVID-19 (e.g., symptoms, preventive measures) [18, 29], enabling safe and effective communication between COVID-19 patients and healthcare professionals [23].

### Effectiveness of digital technologies

A total of 16 studies have applied the quantitative methods to evaluate the effectiveness of digital technologies on mental health, compared to three studies using qualitative methods and one study with mixed methods. Most of the studies have reported the positive effects of digital technologies, either on improving general mental and emotional well-being [13, 23] or addressing specific conditions [19, 22, 28, 32]. Among them, eight included studies examined the effect on depression, while six of them reported statistically significant reductions. Similarity, seven studies examined the effect on anxiety with six reported significant reductions. Based on the findings, both depression and anxiety are the main mental conditions which these digital interventions are more effective on. For instance, Summers et al.’s study [15] indicated that depression and anxiety raised by COVID-19 were significantly decreased after the intervention of a digital behavior change application. Only the telehealth interventions from two studies [16, 18] have resulted in non-significant improvements on mental health among participants.

Among the quantitative studies, web-based survey with pre-post measurement was a popular method used to test the psychological changes associated with the interventions. Mental health outcomes in these studies were measured by validated self-report scales, such as Depression, Anxiety, and Stress Scale [33], Patient Health Questionnaire–9 [34] or UCLA Loneliness Scale [35], which were mainly distributed as an electronic form through the internet. Yet some studies adopted unvalidated measurements which were either author-generated (e.g., a single-item measurement of social resilience [25]) or without clear reference (e.g., scale of concerns about COVID-19 [29]). Only six quantitative [16–18, 20, 26, 31] studies were randomized controlled trials (RCTs) in which the intervention was compared to a control condition such as treat-as-usual [18] or wait-list [17, 20]. Particularly, the comparison from Pizzoli et al. [31] involved three experimental conditions based on different web-based relaxation practices. Due to the limited number of RCTs and diverse outcome measurements, it is currently difficult to estimate the overall quality of evidence. Nevertheless, one RCT [18] did not explicitly report a random component in the sequence generation, which can be considered to have a high risk of bias. Furthermore, due to the heterogeneity of the intervention duration (ranging from seven minutes to eight weeks) and the lack of detail in several studies, no meaningful conclusions can be drawn regarding the optimal exposure to digital technologies required to gain significant mental health improvements.

In contrast, there were three qualitative studies that involved open-ended questions and discussion to explore the effectiveness of digital technologies. Particularly, the results from a social robot intervention [23] were collected from various qualitative field data such as non-participatory and participatory observation, field notes, structured interviews, and even anecdotal records of the digital intervention. These findings have implied that the robot could awaken positive emotions among patients and health-care workers during the COVID-19 pandemic. One study from Ellis et al. [13] adopted mixed-methods to investigate the effects of mobile AR games, but only qualitative results have confirmed the potential role in supporting the mental well-being of players during COVID-19. It further suggested that video games could provide “an escape from the fear accompanying the pandemic”, as well as “aid emotional coping… to alleviate specific mental health conditions” [13].

## Discussion

The on-going COVID-19 pandemic has caused a global health crisis with extraordinary challenges and burdens across many countries, but it also fast-forwards the adoption of digital technologies in health care, particularly the mental health domain. As Wind et al. [7] described, it might be a “black swan” moment when there is major shift in mental health care provision towards e-health and digital care. This scoping review has synthesized the evidence from 20 digital interventions that have been used to support mental health promotion and treatment during the COVID-19 pandemic. They were the timely reflections of current progress in technological solutions for mental health disorders within the COVID-19 context. Through a comprehensive comparison of population focus and type of technology used, the findings serve as potential guidelines for patients and providers to find (or select) innovative solutions that guarantee mental health care during and possibly after the pandemic. While previous relevant reviews only provided narrative description about the potential usage of existing mental health digital interventions for the COVID-19 pandemic [36, 37], the current scoping review is the first review to synthesize recent empirical data regarding the actual mental health effects of digital interventions within the context of COVID-19, thus providing evidence-based recommendations for clinical practice in this emerging domain.

The widespread use of web-based and mobile-based platforms was reported in the review. Mental health services were already overloaded in many places prior to pandemic; but now the pandemic has even brought additional burdens. The telehealth embodiments in web-based interventions, such as audio or video conferencing, are able to overcome the restrictions in traditional face-to-face interactions and be accessible to a large number of people. Particularly, for mobile-based interventions with ubiquitous characteristics, they have a larger capacity in scaling up access and follow-up to mental health services regardless of time and location. Previous studies [38, 39] have already demonstrated the important role of web-based or mobile-based interventions in the response to large-scale risk events. They are effective in delivering mental health support to those experiencing wars, conflicts, and human-caused and natural disasters. Many digital technologies identified in this review have incorporated the telehealth components of remote support or online consultation, indicating a greater utilization of this service delivery mode under the current COVID-19 circumstances.

Nevertheless, a certain number of telehealth services identified in this scoping review were lack of evidence-based therapeutic practices and techniques for the mental health problems occurred in COVID-19. Considering the critical mental health crises raised by the pandemic, the findings have outlined several important problems and challenges of developing customized digital technologies as effective solutions for mental wellness improvement and prevention. As Rauschenberg et al. [36] highlighted in another meta-review, there is a clear need to develop the theoretical foundation of digital interventions through translating good clinical practice standards (such as CBT) into key service components. Furthermore, future success in therapeutic use of digital technologies will also require considerable redesign (i.e., participatory design approaches) with meaningful involvement of both service users and health professionals [37, 40], in order to address the diverse mental health problems related to public health crisis like COVID-19.

There was initial but robust scientific evidence on the effectiveness of digital technologies on mental health outcomes influenced by the COVID-19 pandemic, such as anxiety, stress, depression, or overall mental and emotional well-being. In line with rapid reviews of early literature [9, 36], this scoping review further supported digital technologies to be timely and effective solutions which mitigated the negative psychological impacts of the COVID-19 pandemic. However, considering the limited number of RCTs identified in the review, there is still a lack of rigorous and high-quality evaluation to determine the “true” efficacy of these digital tools within the COVID-19 context, particularly the comparison to routine care involving face-to-face psychotherapy. Meanwhile, since many of the digital technologies have been quickly implemented in this emergency phase, how to maximize their effectiveness is far from known. Many scholars [36, 40] in this field have advocated a blended-care approach with a reasonable balance between face-to-face and tele-platform. A better integration of digital technologies and human interaction may be essential in the post-pandemic phase. In this case, more research is needed to investigate the long-term treatment effects of the integrated interventions with digital and social components, particularly under the global health crisis like COVID-19.

This review identified various subpopulations which digital interventions were assessed on. But there was little discussion on how deployment of digital technologies matched the needs of target subpopulation in the COVID-19 pandemic. Previous studies suggested the demographic factors, such as age, gender, culture, and socioeconomic status, would influence the usage and benefits of digital health technologies [41, 42]. Future studies should continue to address the knowledge gaps in the effective use of digital technologies to deliver appropriate mental care to specific groups during the COVID-19 pandemic. What’s more, the evidence on the use of digital technologies for the vulnerable groups, including older people and COVID-19 patients, is largely limited. It is an important finding since these groups may be particularly challenged by the pandemic [43, 44]. For instance, older adults were reported to suffer the worst physical and mental problems from the pandemic, but obtain the least benefits from these digital solutions [45]. This age group generally has low digital literacy thus might lack sufficient knowledge and skills to fully make of these digital tools. As a result, for health providers and practitioners, they should increase attention and resources to improve digital literacy among the aging population. For technical developers, there is also a need to enhance usability of digital technologies, in order to close the digital divide for mental health care in the long-term.

This scoping review had several limitations. Firstly, although this study reviewed titles and abstracts from nearly 2,000 research articles, it did not include unpublished data or grey literature. We are aware of several emerging evaluations which are not published at academic outlets (e.g., described in news articles or websites). Secondly, only manuscripts published in English were included, thus publication bias is possible in this review. Thirdly, we are unable to perform a meta-analysis due to limited number of RCTs and the diversity across interventions and mental health outcomes. It would be more meaningful to determine the actual effect size if there are sufficient evidence in future studies.

To conclude, the findings of this review demonstrate that digital technologies hold promise in bridging the mental health-care gap during and after the COVID-19 pandemic, when disease news and quarantine measures have terribly threatened public mental health. This study is a timely synthesis of current progress and evaluation which could help with the future planning of digital psychological interventions for various populations and contexts. Further research is needed to conduct more rigorous studies and identify pertinent features of the digital interventions that are likely to achieve more effective mental health outcomes.

## Data Availability

The datasets used and/or analysed during the current study are available from the corresponding author on reasonable request.
